# ANTIDotE: anti-tick vaccines to prevent tick-borne diseases in Europe

**DOI:** 10.1186/1756-3305-7-77

**Published:** 2014-02-21

**Authors:** Hein Sprong, Jos Trentelman, Ingar Seemann, Libor Grubhoffer, Ryan OM Rego, Ondřej Hajdušek, Petr Kopáček, Radek Šíma, Ard M Nijhof, Juan Anguita, Peter Winter, Bjorn Rotter, Sabina Havlíková, Boris Klempa, Theo P Schetters, Joppe WR Hovius

**Affiliations:** 1 The National Institute of Public Health and the Environment (RIVM), Bilthoven, The Netherlands; 2 Academic Medical Center, University of Amsterdam, Center for Experimental and Molecular Medicine, Amsterdam, The Netherlands; 3 Biology Centre of the Academy of Sciences of the Czech Republic (BC ASCR), České Budějovice, Czech Republic; 4 Institute for Parasitology and Tropical Veterinary Medicine, Freie Universität Berlin (FUB), Berlin, Germany; 5 Center for Cooperative Research in Biosciences (CIC bioGUNE), Derio, Spain and Ikerbasque Foundation, Bilbao, Spain; 6 GenXPro GmbH (GenXPro), Frankfurt am Main, Germany; 7 Institute of Virology, Slovak Academy of Sciences (IV SAS), Bratislava, Slovak Republic; 8 Merck Sharp & Dohme Animal Health, Boxmeer, The Netherlands

**Keywords:** *Ixodes ricinus*, Vaccine, Lyme borreliosis, Tick-borne encephalitis, Babesiosis, Public health

## Abstract

*Ixodes ricinus* transmits bacterial, protozoal and viral pathogens, causing disease and forming an increasing health concern in Europe. ANTIDotE is an European Commission funded consortium of seven institutes, which aims to identify and characterize tick proteins involved in feeding and pathogen transmission. The knowledge gained will be used to develop and evaluate anti-tick vaccines that may prevent multiple human tick-borne diseases. Strategies encompassing anti-tick vaccines to prevent transmission of pathogens to humans, animals or wildlife will be developed with relevant stakeholders with the ultimate aim of reducing the incidence of tick-borne diseases in humans.

## Background

### Tick-borne diseases as a public health concern in Europe

*Ixodes ricinus* is a hard tick that transmits a variety of pathogens of medical and veterinary importance. These include *Borrelia burgdorferi* sensu lato, *Borrelia miyamotoi*, *Anaplasma phagocytophilum*, *Rickettsia* spp*.*, *Babesia* spp*.*, and tick-borne encephalitis virus (TBEV). The most prevalent tick-borne infection of humans in Europe is Lyme borreliosis, with at least 65,000 documented cases yearly [1]. The most pathogenic infection is caused by TBEV, with 1 to 2% of patients reportedly dying from the European subtype [2], and with up to 46% of TBE patients suffering from long-term sequelae [3]. Infection of most other tick-borne pathogens associated with *I. ricinus*, such as *Babesia* spp., but also *B. miyamotoi*, is usually mild and may easily go undiagnosed. However, infection of immunocompromised individuals can lead to a progressive and more severe illness [4,5]. A relatively low number of severe human babesiosis cases have been reported in literature, which is surprising considering the widespread distribution of *Babesia*-infected ticks and the size of the population at risk [6].

The incidences of Lyme borreliosis and tick-borne encephalitis are on the rise in several European countries [1,2,7] and diseases caused by other pathogens, such as *Neoehrlichia mikurensis*, and *B. miyamotoi* are clearly emerging [5,8]. Environmental (e.g. climate change and landscape management), socio-economic and demographic factors (e.g. population aging and life-styles) synergistically increase the risk of acquiring tick-borne diseases [9-13]. Based on these findings, the European Center for Disease Prevention and Control has predicted that the incidence of tick-borne diseases will rise in the near future [14]. Therefore, the old adage ‘prevention is better than cure’ also holds true for tick-borne diseases.

### Current control measures

Conventional control measures on tick-borne zoonoses, such as the widespread application of detrimental acaricides to control ticks and the culling of wildlife reservoirs, are becoming more and more socially unacceptable [15]. Where the risk of infection is high, and/or the resulting disease severe, vaccines may be the most efficient and cost-effective means of prevention and control [16]. Anti-tick vaccines targeting other tick species already exist and are being used in the veterinary field. The strategy behind these vaccines is to locally control *Rhipicephalus* (*Boophilus*) tick species, and act as a safe and environmentally friendly alternative to acaricides [17,18]. Application of anti-tick vaccines was shown to dramatically decrease the incidence of bovine babesiosis [19]. Whether anti-tick vaccines can also be used to (locally) eradicate *I. ricinus*, and prevent human tick-borne diseases remains to be established. It may not prove to be a easy challenge due to the very large host range of *I. ricinus* and its ability to survive in a variety of habitats, including deciduous and coniferous woodlands, heathlands, moorlands, rough pastures, forests, urban parks, and even gardens [20].

Vaccines against TBEV are available, but require multiple doses and frequent boosters to induce and maintain immunity [21] and the vaccination coverage in many Central and Eastern Europe is remarkably low [22]. A vaccine protecting against Lyme borreliosis is unavailable, but a refined vaccine has recently been tested in a Phase I/II trial [23]. Based on the experiences with the previous Lyme vaccine that was on the American market from 1998 to 2002, it remains to be seen whether a Lyme vaccine will be widely used [24]. There is no human vaccine to prevent babesiosis or any other of the other above-mentioned pathogens. Ideally, one would like to have a single vaccine (for humans), protecting against multiple tick-borne diseases.

### The idea behind ANTIDotE

Rather than developing vaccines against all the pathogens transmitted by *I. ricinus*, one vaccine targeting the tick itself could have the same effect by blocking the transmission of *any* pathogen. The idea of such a vaccine is supported by the observation that multiple exposures of certain mammalian species to tick bites results in an inability of ticks to successfully take a blood meal, a phenomenon designated as ‘tick immunity’ [25,26]. *Ixodes* ticks not only introduce pathogens, but also salivary proteins into the host skin that facilitate their feeding and even the transmission of pathogens by modulating host defense mechanisms. Anti-tick immune responses are based on both cellular and humoral immune responses directed against selected tick (salivary gland) antigens. Of paramount importance, ‘tick immunity’ impairs tick feeding, but also protects animals from the development of experimental infections with tick-borne pathogens [27-29]. These anti-tick immune responses can be simulated by vaccination of laboratory animals with tick salivary gland extract or selected tick salivary gland proteins [30]. In addition, targeting tick salivary proteins that facilitate the transmission of pathogens from the tick to the host by (neutralizing) antibodies may also prevent successful pathogen transmission, without affecting the tick’s ability to feed [31-33]. Notably, humans with hypersensitivity to tick-bites are also at a lower risk of contracting Lyme borreliosis [34]. In other words, the identification and characterization of tick salivary gland proteins involved in feeding and transmission of bacteria, viruses and protozoa will greatly facilitate the development of an anti-tick vaccine that could prevent multiple human tick-borne diseases. Such a vaccine could either be used in humans directly, or in animals and wildlife in order to indirectly reduce the risk of contracting tick-borne disease for humans.

### The ANTIDotE consortium and project

#### The ANTIDotE consortium

**Figure 1:**
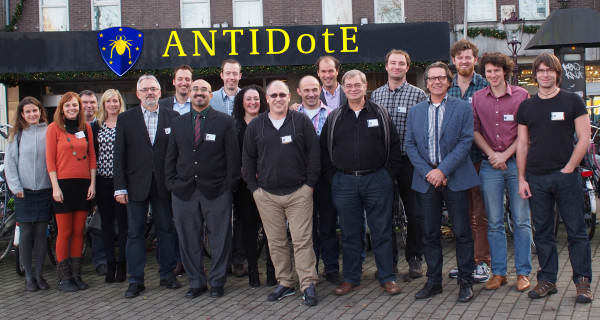
**The participants of the official launch meeting of ANTIDotE.** This meeting was held in Amsterdam, The Netherlands, in December 2013.

ANTIDotE is the acronym of the project “*Ant*i-tick Vaccines to Prevent *Ti*ck-borne *D*iseases in *E*urope. It is also the name of the consortium that will push forward the project. The consortium consists of seven European institutes funded by the European Commission through the Seventh Framework Program for Research (FP7-Health). The consortium is headed by Dr. Joppe Hovius, Principal Investigator at the Academic Medical Center in Amsterdam, and further consists of scientists from the Institute of Parasitology at the Biology Centre of the Academy of Sciences of the Czech Republic, the Institute for Parasitology and Tropical Veterinary Medicine of the Freie Universität Berlin, Center for Cooperative research in Biosciences from Spain, the medium-sized enterprise GenXPro from Germany, the Institute of Virology of the Slovak Academy of Sciences, and Dutch National Institute for Public Health and Environment. It brings together a unique combination of industrial, research and public health institutes with ample research experience on ticks, anti-tick immune responses, transmission of tick-borne pathogens, anti-tick vaccines and surveillance and control of vector-borne diseases. The official launch meeting was held on December 1st, 2013 in Amsterdam (Figure 1).

#### The ANTIDotE project

**Figure 2:**
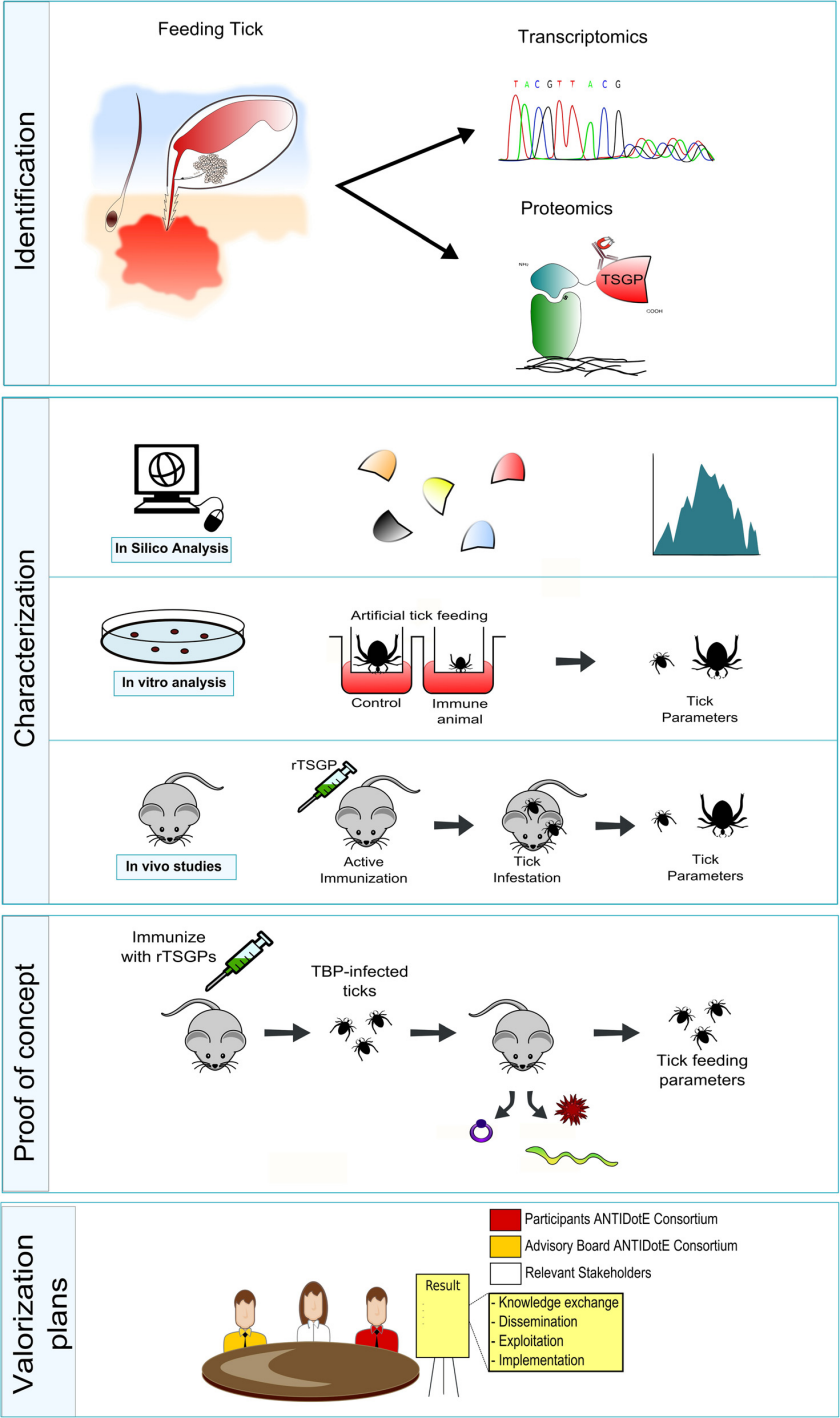
**The ANTIDotE approach.***Identification*: Using state of the art proteomic and transcriptomic approaches we will identify novel tick salivary gland proteins. *Characterization*: These novel tick salivary gland proteins will be characterized using *in silico*, *in vitro* and *in vivo* techniques. *Proof of concept*: Promising candidates will be assessed in a proof of concept study as anti-tick vaccines to protect against tick-borne diseases. Both transmission as well as tick feeding parameters will be assessed. The symbols (from left to right) represent *Babesia*, *Borrelia* nd TBEV, respectively. *Valorization plans*: Through an integrated and multidisciplinary approach involving European public health institutes, health organizations and industrial companies, we will examine how to develop anti-tick vaccines and implement these in public health systems in Europe.

ANTIDotE’s key objective is to identify antigens from *I. ricinus* that could serve as candidates for an anti-tick vaccine, which is able to protect against multiple European tick-borne diseases (Figure 2). Using state of the art proteomic and transcriptomic approaches we will identify and characterize novel tick salivary gland proteins, which will be subsequently assessed as anti-tick vaccines to protect against Lyme borreliosis, TBE and babesiosis in animal models. Importantly, this is not where ANTIDotE stops. We will also explore ways to expedite and facilitate future utilization and implementation of anti-tick vaccines in public health systems [35]. Novel anti-tick vaccines could be used in humans and finding a candidate that holds that potential and protects against multiple human tick-borne diseases is ANTIDotE’s main ambition. However, as an alternative approach, anti-tick vaccines that interfere with tick feeding and/or pathogen transmission could also be adapted for use in domesticated animals or wildlife. This could (locally) decrease tick or pathogen populations in the environment, which reduces the risk of contracting tick-borne diseases for humans. The application of vaccines in domesticated animals or wildlife to prevent human disease is a challenge and there are only a few practically implemented precedents [16]. Therefore, through a multidisciplinary and integrated approach with several European public health institutes, in collaboration with relevant stakeholders, such as the European health organizations and end users, the ANTIDotE consortium will examine if and how anti-tick vaccines can be incorporated in health systems in Europe. We will do so by organizing two workshops during the five-year course of the project, at which the different stakeholders (e.g. scientists, clinicians, public health experts, and interested industrial partners) will be invited. For more information we would like to refer to the website http://www.antidote-fp7.org and interested institutes or individuals are encouraged to contact the leader of the specific work package, Dr. Hein Sprong, or the ANTIDotE coordinator, Dr. Joppe WR Hovius.

## Abbreviations

(r)TSGP: (recombinant) Tick salivary gland protein; TBP: Tick-borne pathogen.

## Competing interests

The authors declare that they have no competing interests.

## Authors’ contributions

All authors contributed to the manuscript and approved with the final version.
